# Estimating Plasma Glucose from Interstitial Glucose: The Issue of Calibration Algorithms in Commercial Continuous Glucose Monitoring Devices

**DOI:** 10.3390/s101210936

**Published:** 2010-12-03

**Authors:** Paolo Rossetti, Jorge Bondia, Josep Vehí, Carmine G. Fanelli

**Affiliations:** 1 Instituto Universitario de Automática e Informática Industrial, Universidad Politécnica de Valencia, Camino de Vera, s/n, 46022 Valencia, Spain; E-Mail: jbondia@isa.upv.es; 2 Dipartimento di Medicina Interna, Scienze Endocrine e Metaboliche, Università degli Studi di Perugia, Perugia, Italy; E-Mail: cgfanelli@interfree.it or cgfanelli@unipg.it; 3 Institut d’Informatica i Aplicacions, Universitat de Girona/Campus Montilivi, Girona, Spain; E-Mail: josep.vehi@udg.edu

**Keywords:** continuous glucose monitoring, calibration algorithms

## Abstract

Evaluation of metabolic control of diabetic people has been classically performed measuring glucose concentrations in blood samples. Due to the potential improvement it offers in diabetes care, continuous glucose monitoring (CGM) in the subcutaneous tissue is gaining popularity among both patients and physicians. However, devices for CGM measure glucose concentration in compartments other than blood, usually the interstitial space. This means that CGM need calibration against blood glucose values, and the accuracy of the estimation of blood glucose will also depend on the calibration algorithm. The complexity of the relationship between glucose dynamics in blood and the interstitial space, contrasts with the simplistic approach of calibration algorithms currently implemented in commercial CGM devices, translating in suboptimal accuracy. The present review will analyze the issue of calibration algorithms for CGM, focusing exclusively on the commercially available glucose sensors.

## 1. Introduction

Following the demonstration of the causal relationship between microvascular complications and hyperglycaemia [1,2], monitoring of glycaemic status is considered a cornerstone of diabetes care. During the past 40 years, technical advances have allowed for dramatic changes of monitoring of metabolic control. Before 1975, routine patient monitoring consisted of urine glucose and ketone determinations [3]. Typically, physicians monitored occasional laboratory blood glucose determinations and reviewed patient home urine testing records. The primary purpose of monitoring was to provide information to the patient’s health care provider to guide changes in therapy to relieve symptoms due to hyperglycemia (polyuria, polydipsia and nocturia) rather than to achieve specific glycaemic goals. Since 1975, technical advances allowed for radical changes in glucose monitoring. In 1971 the first blood glucose monitor for point-of-care use in patients with diabetes was patented in the USA by Anton Clemens: the Ames Reflectance Meter. It was based on optical detection of a color change on glucose oxidase-based strips, and was succeeded by the Ames Eytone, which became commonplace in physicians’ offices and hospitals. A number of clinical studies, in the late 1970s, demonstrated that the technology improved metabolic control and was applicable for self-management of patients in everyday life [4–8]. During the 1980s, technology for home glucose measurement moved from reflectance devices to electrochemical biosensor-based glucose sensing, the first amperometric glucose biosensor being described in 1984 and commercialized in 1987 [9,10]. Due to technical advances and to the increasing evidence of its usefulness, by the mid-1980s, self-monitoring of blood glucose (SMBG) had already replaced urine glucose testing as the recommended method of home testing and, at present, it is considered a fundamental part of the management of all patients with diabetes, especially those who use insulin [11].

Parallel to the development of SMBG, progress with enzyme electrodes in the 1970s [12–16] allowed for the emergence of continuous glucose monitoring (CGM), and for the subsequent development of the first prototypes of glucose-sensor controlled insulin infusion systems, by different groups [17–20]. The next two decades saw huge progress in the development of continuous glucose sensing. Research focused on the skin as an appropriate candidate for direct glucose measurement. Indeed, the subcutaneous tissue is easily accessible for sensor implantation and measurement of glucose in the interstitial fluid, with fewer problems as compared to the intravascular space. The amperometric glucose-sensing technique was refined and this process culminated, in 1999, with the development and FDA approval of the CGMS^®^, the first commercial CGM device [21]. In the attempt to obtain non-invasive CGM, several technologies alternative to electroenzymatic sensors have been studied or are under development: spectroscopy, sonophoresis, polarimetry, infrared, fluorescence, light-emitting diode, electromagnetic radiant ray and laser [22]. However, to date, direct interstitial fluid glucose measurement is the only technique that has been thoroughly tested and is commercially available for diabetic people. Devices using this technique are referred to as minimally invasive, and operate with either a subcutaneous needle-like sensor, sensor-based microdialysis or reverse iontophoresis. All of them use glucose-oxidase enzyme-based technology and differ in the way the interstitial fluid is sampled.

Evaluation of metabolic control, as well as adjustment of diabetes therapy, has been classically performed based on measurement of glucose concentrations in blood samples. However, devices for CGM measure glucose concentration in compartments other than blood, *i.e.*, directly in the subcutaneous interstitial fluid (commercially available devices), or indirectly from changes in specific properties of a given tissue (usually the skin: non-invasive methods under development). This means that CGM requires calibration against concurrent blood glucose values, thus providing an estimate of blood glucose concentration, based on the assumption that glucose concentration in the interstitial fluid (or in the skin, in the case of non-invasive methods), is directly related to blood glucose concentration. However, this is a simplification, and the accuracy of the estimation of blood glucose from the measurement in the interstitial space will depend, among other factors, on the calibration algorithm. Indeed, the latter is a function that, in some way, includes the relationship between plasma and interstitial glucose. Clearly, the more precise and robust the calibration algorithm, the plasma glucose estimates will be more accurate. Unfortunately, however, few studies have systematically investigated the relationship between plasma and interstitial glucose [23–29], with heterogeneous results. This highlights the complexity of such a relationship, which contrasts with the rather simplistic approach of calibration algorithms currently implemented in commercial CGM devices, resulting in suboptimal accuracy especially under conditions of hypoglycemia [22].

The present review will analyze the issue of calibration algorithms for CGM. For the sake of brevity, we will focus exclusively on the commercially available glucose sensors. In 2007, the only device using the iontophoresis technique, the Glucowatch Biographer (Animas Corp, West Chester, PA, USA) was retired from the market. The GlucoDay (Menarini Diagnostics, Florence, Italy), a microdialysis device, is intended only for professional use. Therefore, only needle-like subcutaneous sensors are available on the market for home CGM. They are summarized in Table 1.

## 2. Glucose Sensing from the Interstitial Space

Current commercial glucose sensors are all based on the indirect measurement of glucose from the interstitial space through amperometric enzyme electrodes based on glucose-oxidase (GOx). Good reviews of these systems, as well as other techniques under investigation for non-invasive measurement, can be found elsewhere [30–32].

The operating principle of amperometric sensors is the measurement of the current flowing from an oxidation reaction, at a working electrode, to a reduction reaction, at a counter electrode [33]. To this purpose, a potential is applied between the working electrode and a reference electrode. Three electrodes are thus needed (working, counter and reference electrodes), although some sensors use a two-electrode configuration (working and counter-reference electrode), combining the counter and reference electrodes. Medtronic and Abbott use three-electrode configurations. DexCom uses a two-electrode configuration.

In the case of glucose sensing, GOx is immobilized at the working electrode. GOx catalyses the oxidation of glucose to gluconolactone. To this end, GOx requires as cofactor Flavin Adenine Dinucleotide (FAD) that will act as electron acceptor reducing to FADH_2_, according to the following reaction [32]:

(1) $$\text{Glucose}\;+\;\text{GOx}\left(\text{FAD}\right)\to \;\text{Gluconolactone}\;+\;\text{GOx}\left({\text{FADH}}_{2}\right)$$

The FAD cofactor (redox active center) is deeply embedded in the GOx molecular structure [34]. This necessitates the use of mediators or other strategies to improve communication between the enzyme and the electrode surface “guiding” electrons to the electrode. The natural mediator is the couple oxygen/hydrogen peroxide (O_2_/H_2_O_2_), according to the reactions:

(2) $$\begin{array}{l} \text{GOx}\left({\text{FADH}}_{2}\right)+{\text{O}}_{2}\to \text{GOx}\left(\text{FAD}\right)+{\text{H}}_{2}{\text{O}}_{2}\\ {\text{H}}_{2}{\text{O}}_{2}\to 2{\text{H}}^{+}+{\text{O}}_{2}+2{\text{e}}^{-} \end{array}$$

The flavin is re-oxidized in the presence of oxygen, producing hydrogen peroxide. This is monitored measuring the current generated after the application of a potential (around +0.6 V *vs*. Ag/AgCl) between the working electrode and a reference electrode. This is the method used, for instance, in the Medtronic and DexCom monitors.

Two main problems have to be dealt with:

- Other electro-active molecules such as uric acid and ascorbic acid may interfere in the measurement, depending on the potential applied. To reduce interference and increase selectivity to glucose, membranes limiting the access of these molecules to the electrode surface are included, or electrodes are built in materials requiring a lower potential.
- Glucose concentration is much higher than oxygen concentration. A proper glucose-oxygen ratio must be obtained. To this end, membranes are included limiting the transport of glucose to the electrode in order to maximize oxygen availability.

The main differences between the Medtronic and DexCom sensors consist in how these problems are tackled. The Medtronic sensor uses a polymer membrane to address the oxygen deficit problem. In DexCom sensor a barrier membrane is incorporated to reduce the glucose flow, reducing consequently the production of hydrogen peroxide, which may damage the electrode. In this way, more durability is gained [32].

The Abbott sensor avoids the use of oxygen as a mediator. Instead, a wired enzyme technology is used. The GOx active center is “wired” to the electrode by means of osmium-based redox polymers, establishing direct electrical communication [35]. This allows a low reaction potential (−0.2 V), reducing the interference of electro-active molecules [33].

## 3. Relationship between Plasma, Capillary and Interstitial Glucose

**Figure 1.**
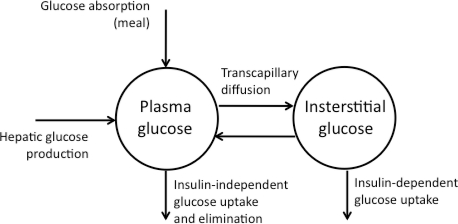
Compartmental model of glucose transport.

Glucose exchange across the capillary walls occurs by simple diffusion across a concentration gradient. However, this process is not instantaneous and appears to be influenced by both blood flow and capillary permeability [36,37]. Indeed, recent data demonstrate that the interstitial glucose pool of muscle and adipose tissue are part of a compartment which is in relatively slow equilibrium with plasma glucose (see Figure 1) [38].

The obvious consequence of limited transcapillary exchange of glucose is that any change in plasma glucose concentration, as well as in the metabolic rate and glucose uptake by cells, will affect the plasma-to-interstitial fluid (ISF) concentration difference [23,24,26,28,39]. The latter is best known as physiological lag time between plasma and ISF glucose. The presence of such a lag is widely reported and acknowledged. However, a great degree of variation has been observed, with lag times ranging from 0 to 45 minutes. Although the majority of researchers in the field put the lag time into the 5–15 minutes range [27,40–46], the huge variability observed is well explained by the complexity of the plasma to ISF glucose relationship. Indeed, different experimental conditions, representative of different metabolic conditions, are likely to result in different estimations of physiological lag time. As an example, the fall of glucose concentration in the interstitium has been shown to occur either before [26,28] or after [47,48] that observed in plasma. This may be explained, at least in part, by different plasma insulin concentrations reached during the experiments. In fact, in physiology, glucose fluxes (*i.e.*, hepatic production and peripheral uptake), are strictly insulin dependent. This means that, under low (but relatively high) plasma insulin concentrations, hypoglycemia is only the result of the suppression of hepatic glucose production (EGP): in this case, glucose concentration is expected to fall sooner in plasma than in the ISF. On the other hand, under high insulin concentrations (such as following prandial insulin administration), hypoglycemia is the result of both hepatic suppression and the increase of peripheral uptake of glucose by fat and muscle (GU): this scenario is more complex (the so called ‘push-pull phenomenon’) and glucose concentration may fall in ISF sooner than in plasma [23,26,28]. Several recent studies that have evaluated the plasma-to-ISF relationship, have used correlation analysis to assess the lag time, underlines the small impact of the physiological delay on the overall analytical error of CGM monitors as compared to reference methods [27,40–46]. In particular, one study specifically argues against the existence of a push-pull phenomenon [46], suggesting that the plasma-to-ISF glucose dynamic may be simpler, and the delay of interstitial glucose upon plasma glucose change may be smaller, than previously postulated [23,38]. Those studies are largely based on data from clinical trials. However, if the latter are representative of real life for patients with diabetes, they are not best suited to describe physiology. Indeed, pooling of data from uncontrolled and potentially different metabolic conditions may easily mask physiologic phenomena. Another potential simplification of the plasma-to-ISF glucose relationship may be due to the use of correlation or curve fitting methods (maximum statistical agreement criterion), to assess time lag. Indeed, if these methods can estimate the mean delay (which is described as a point estimate) between the two compartments, they are based on the assumption that its relationship is linear. However, this may not be true, as supported by the finding of different time lags at different rates of change of plasma glucose [41,43] and by previous elegant physiologic studies [23], even with independent measurement of ISF glucose concentrations [26]. Hence, accurate estimation of plasma glucose from measurements in the ISF seems to require mathematical models describing the relationship (tissue-specific) between plasma and ISF glucose levels, both during steady state and dynamic conditions. However, to make the issue even more complex, there is the potential influence of the technique used to sample the ISF on the lag time. Indeed, it must be taken into account that insertion of a needle-like sensor, as well as a microdialysis fiber or a microperfusion catheter, disrupts the physiologic architecture of the subcutaneous tissue and is associated with an inflammatory response [49] and a foreign body reaction, which are specific to the materials used [50]. Indeed, the biocompatibility of implantable glucose sensors remains a critical issue in limiting CGM device longevity and functionality, and most functional loss of biosensor activity is assumed to be caused by histological changes that occur in the tissue surrounding the implant (inflammatory reaction and/or fibrous encapsulation) [50]. The material-tissue interaction during sensor implantation, the so-called biofouling, is recognized as one of the major factors resulting in unpredictable and unexplainable behaviors of implanted glucose biosensors. In fact, the performance of implanted biosensors may greatly benefit from the use of more biocompatible outermost coatings, as recently demonstrated in animal experiments where a hydrogel coating was effective in minimizing tissue reactions surrounding implanted minimally invasive needle-type glucose biosensors [51]. Improving glucose sensors biocompatibility would rule out the problem of loss of sensitivity during the sensor lifetime [52], certainly allowing for better description of the plasma to interstitial glucose dynamic.

Finally, it should be noted that the intra-individual sensor to sensor variability (two identical sensors placed in the same subject at the same time), has been shown to be greater than the apparent physiological lag time [27], potentially hampering our ability to describe physiologic phenomena. This underlines the importance of finding calibration strategies which include dynamic models specific to each device used for CGM.

## 4. Estimating Plasma Glucose from Interstitial Glucose

### 4.1. Getting Calibration Points

Calibration of CGM devices is usually done by means of plasma glucose values obtained with glucometers, which measure capillary plasma glucose concentrations. This is reasonable, since self monitoring of capillary plasma glucose (SMBG) is the gold standard of glycemic testing in everyday life diabetes care. However, it may contribute substantially to the inaccuracy of CGM devices, as has been recently demostrated by Kamath *et al*., who showed a significant reduction of CGM error when it was calibrated against a reference method (venous plasma glucose with the YSI) instead of capillary plasma glucose [42]. This is not surprising, since accuracy of SMBG is influenced by several factors such as test strip handling, proper glucometer coding and procedural factors (meter cleanliness and careless hand washing, the size and placement of the blood sample, *etc*.), among others (for a detailed description see reference [53]). Indeed, accuracy of SMBG has been demonstrated to be technique dependent [11], as shown by poorer performance of glucometers among patients as compared with technicians [54]. In addition, the relationship between venous, plasma and capillary blood measurements is not fixed, varying with the metabolic status of the patient: if they are similar in the fasting state, post-prandial capillary samples generally show values higher than in venous plasma [55,56]. To generate further confusion, some glucometers quantify whole-blood glucose (instead of the recommended plasma glucose), which reads 10%–15% lower than plasma. All these explain why today’s meters are capable of producing results that meet established standards of accuracy under controlled conditions, but clinical studies demonstrating consistently comparable performance in the hands of patients are lacking.

Given the abovementioned issues on calibration with SMBG values, it is not surprising that both the quality and the timing of calibration points have been recognized as crucial factors influencing the accuracy of CGM readings [57,58]. However, the importance of calibrating during steady state conditions (*i.e.*, avoiding calibration during rapid changes of plasma glucose) has been recently questioned by some authors, who showed no detrimental effect [42], or even improvement, in CGM accuracy following calibration under dynamic conditions [59], demonstrating that reduction of the patients’ SMBG technique related errors is probably of greater effectiveness. In this regard, Choleau *et al.* demonstrated the effects of the errors in the measurement of capillary blood glucose on the accuracy of blood glucose estimations and showed how they depend on the calibration algorithm used for blood glucose estimation (one-point *versus* two points calibration procedure) [60,61]. These aspects should be taken into account when calibrating CGM sensors and when the accuracy of different CGM devices is compared.

### 4.2. Principles of Calibration Algorithms in Commercial CGM Devices

In this section, principles of calibration algorithms implemented in the continuous glucose monitors currently on the market are described. All the information contained here has been extracted from issued patents and published patent applications. For an extensive review of real-time calibration algorithms, filtering and alarms see [62].

The first algorithm described here corresponds to the one implemented in the Medtronic CGMS Gold [63,64]. The algorithm is intended to estimate blood glucose from raw intensity measured in the interstitial fluid in a retrospective way. This is to say, after collecting all the data corresponding to three days of operation of the sensor, the algorithm tries to adjust the estimation of blood glucose to minimize the absolute relative error of this estimate with respect to the capillary blood glucose in the calibration points. Once the stabilization process is completed, the glucose monitor measures the continuous electrical current signal (ISIG) generated by the glucose sensor at a sampling rate of 10 seconds. At an interval rate of one minute, highest and lowest values are discarded and the remaining 4 values averaged. Every five minutes, the highest and lowest of those values are ignored and the average of the remaining three values is stored. Clipping limits are applied to reduce the effects of outliers, transients or extraneous data. Each memory storage value is considered valid unless a cancellation event occurs, and the signal is advanced in time two sample periods (10 minutes) to account for the physiological lag between plasma and interstitial fluid glucose, *i.e.*, intensity 10 minutes ahead (Valid ISIG) is considered for glucose prediction.

The single point calibration algorithm is based on the assumption that the Valid ISIG will be 0 when blood glucose is 0. The Single Point Sensitivity Ratio (SPSR) is calculated as the slope of a paired calibration point:

(3) $$\text{SPSR}=\frac{\text{Blood}\;\text{Glucose}\;\text{Reference}\;\text{Reading}}{\text{Valid}\;\text{ISIG}}$$

If SPSR is less than a sensitivity threshold value, then a modified SPSR (MSPSR) is calculated using the offset value:

(4) $$\text{MSPSR}=\frac{\text{Blood}\;\text{Glucose}\;\text{Reference}\;\text{Reading}}{\left(\text{Valid}\;\text{ISIG}-\text{Offset}\right)}$$

Therefore, the calibrated blood glucose level is:

(5) Blood Glucose Level=(Valid ISIG−Offset)*MSPSR

Offset value is usually determined empirically.

When more than one paired data is available, single point calibration is augmented using a modified linear regression technique. The linear regression equation is:

(6) $$\text{MLRSR}=\frac{\sum_{i=1}^{N}\left[\left(X_{i}-\text{Offset}\right)Y_{i}\right]}{\sum_{i=1}^{N}\left[{\left(X_{i}-\text{Offset}\right)}^{2}\right]}$$

where *X_i_* is the i-th Valid ISIG of paired calibrations data points, *Y_i_* is the i-th Blood Glucose Reference Reading of paired calibration data points and N is the total number of paired calibration data points.

In retrospective algorithms, given a new Valid ISIG, MLRSR is calculated for the data calibrations pairs in a window of 24 hours (12 hours before and 12 hours after, including at least three calibration data pairs) using several offset values (empirically chosen). The applied slope corresponds to the MLRSR that minimizes the MAD (Mean Absolute Difference) of the calibrating data pairs within the time window. Some refinements are included to smooth the estimation of blood glucose when offset changes.

The real-time algorithm used by Medtronic [65] is based on the same principle of the retrospective one, with some small changes. As only data from previous time instants are available, the linear regression equation is modified and the linear regression technique described above is executed using four paired calibration data points, the most recent and points from 6, 12 and 18 hours prior. Real time calibration adjustment is performed to account for changes in the sensor sensitivity during the lifespan of the glucose sensor. In these algorithms, when a new blood glucose reference is obtained, a calibration factor current (CFc) is calculated (CFc = Meter BG/current ISIG value). The CFc should meet some criteria to accept a new current value as accurate ISIG.

In a more robust formula for approximating the slope, more recent ISIGs are given more weight than older ones:

(7) $$\text{Filtered}\;{\text{ISIG}}_{\left(i\right)}=\frac{\sum_{j=i-\left(n-1\right)}^{i}\omega_{j}\cdot \text{Raw}\;{\text{ISIG}}_{j}}{\sum_{j=i-\left(n-1\right)}^{i}\omega_{j}}$$

Regarding time lag, the procedure followed in the retrospective case is no longer feasible. In real time algorithm, Wiener filters are used to predict values in the future, although no details are given by the manufacturer. In a recent patent application [66], other adaptive filters, such as the Kalman filter, are proposed for better estimation and prediction of plasma glucose.

DexCom continuous glucose monitors [67], use a linear least squares regression performed in the initial calibration set to create a conversion function. Regression calculates a slope and an offset, which defines the conversion function: y = mx + b, where x-axis represents blood glucose and y-axis represents sensor data. To account for changes in sensitivity [68], the analyte sensor is provided with an auxiliary electrode. For example, the change in sensitivity is measured by measuring a change in oxygen concentration, which can be used to provide an independent measurement of the maturation of the biointerface, and to indicate when recalibration of the system may be advantageous.

The auxiliary working electrode can be configured to measure the baseline of the glucose sensor over time. The baseline signal can be subtracted from the glucose signal obtained from the glucose-measuring working electrode to obtain the signal contribution due to glucose only according to the following equation:

(8) $${\text{Signal}}_{\text{glucose}\;\text{only}}={\text{Signal}}_{\text{glucose}-\text{measuring}\;\text{working}\;\text{electrode}}-{\text{Signal}}_{\text{baseline}-\text{measuring}\;\text{working}\;\text{electrode}}$$

This leads to a simplified calibration technique, wherein variability in the baseline has been eliminated. Calibration of the resultant differential signal can be performed with a single matched data pair by solving the equation y = mx. With regard to time lag compensation, regression techniques are used to predict values 15 minutes ahead in time.

The third continuous glucose monitor currently on the market, Abbott’s Freestyle Navigator^®^, bases its basic calibration algorithms on calculating weighted sensitivities [69], as in Medtronic’s monitors. To account for the estimation of sensor sensitivity [70], the analog interface is configured to provide a perturbation control signal that affects the sensor response, as an example, changing the voltage level that is applied to the sensor between the work and reference electrodes. The sensitivity estimation may be determined based on the difference in measured response to different voltage levels according to a lookup table. Based on the measured response to the perturbation control signal, the sensor parameters are estimated and thus blood glucose level calculated. This procedure can be repeated continuously. A method to include lag compensation on the measured data that is used to update the calibration parameter is also included in the calibration algorithms [71]. To do that, some filters (IIR, FIR, Kalman filters, *etc*.) are used to determine the rate of change of the monitored data at the calibration time. Using the glucose rate at time T, the lag corrected sensor data at T-1 can be determined and then the calibration parameter updated. Finally, the lag corrected calibrated sensor data at time T is determined.

In summary, all the calibration algorithms implemented in commercially available home CGM devices are based on linear regression techniques. Some differences exist in the strategies adopted for compensation for changes in sensor sensitivity, as well as for lag time. However, regarding the latter issue, not many technical details are provided by the manufacturers, jeopardizing the comparison between algorithms. Main characteristics of current real-time calibration algorithms are reported in Table 2.

### 4.3. Limitations of Current Calibration Strategies and Future Trends in Calibration Algorithms

Table 3 shows the accuracy reported for the latest monitors from Medtronic, DexCom and Abbott. Although no exact implementation of the calibration algorithms is disclosed, it is considered here that the principles described in the corresponding patents are followed. It is observed that differences are small, despite the differences in sensing technology and calibration algorithms. A slightly better accuracy is observed for the Abbott FreeStyle Navigator, which uses wired enzyme technology *vs*. oxygen mediator as in the case of Medtronic and DexCom. However it is not possible to know whether this has significance in the achieved accuracy improvement, or whether it is due to the calibration algorithm itself. Medtronic Paradigm VEO and DexCom SEVEN Plus have the same mean ARD, despite the apparently more sophisticated methodology for the compensation of sensitivity changes by DexCom. However, the gold standard used for the calculation of the ARD was different and this may bias the results. When compared to a previous monitor by Medtronic, the Guardian REAL-Time, with the same gold standard, an improvement of 4% in the mean ARD is achieved by the DexCom algorithm.

Thus, the calibration algorithm seems to have relatively little impact on a monitor’s accuracy. One explanation is that errors in reference measurements from SMBG, leading to substantial bias in the calibrated CGM signal [42], mask the effect of different calibration algorithms. However, another possible reason is that all current algorithms are based on simple linear regression techniques. A static relationship between the measured intensity and plasma glucose is considered in this way, neglecting any plasma-interstitium transport dynamics. In fact, usually the time lag between plasma and interstitial glucose (and thus to sensor intensity signal) is neglected by the calibration algorithm and calibration points are recommended to be taken at “stationary” metabolic states where equilibrium between plasma and interstitial glucose is expected [62]. Indeed, recent data demonstrate that the use of dynamic models in the calibration algorithm for the estimation of plasma glucose from the sensor-supplied intensity signal, instead of static linear regression, allows for a significant accuracy improvement, even with the use of population model parameters [75]. In this work, a population autoregressive third order model was tuned from intensity measurements given by a Medtronic CGMS Gold monitor and reference plasma glucose measurements (Beckman Glucose Analyzer). Predictions given by the model were corrected at every calibration point introduced by the patient. In particular, a cross-validation analysis yielded an overall mean ARD of 9.6%, and 8.1% in the hypoglycemic range, substantially improving currently reported data of accuracy under hypoglycemic conditions [47]. The sensitivity and specificity with respect to hypoglycemia detection were 91.5% and 95%.

Characterization of the changes in transport dynamics due to the metabolic state may also be of special significance. In a recent study by our group (results not yet published) local model techniques have revealed the need for different dynamic models (in this case first order linear models) in the different phases of a hypoglycemic clamp consisting of a glucose decrement to hypoglycemia, a hypoglycemia plateau, and a glucose increment to hyperglycemia. A mean ARD of 7.28% was achieved when compared to gold standard plasma measurements, with 98.46% of glucose estimations fulfilling the ISO criteria (15 mg/dL error for glucose values below 75 mg/dL and 20% error otherwise).

A different approach was adopted by Kuure-Kinsey *et al*. who used a Kalman filter to improve CGM accuracy [76]. In particular, they developed a dual-rate Kalman filter which used the information from both the frequent sensor measurements and the infrequent fingerstick measurements, demonstrating superiority over the one-point calibration method. However, this algorithm still neglected the blood glucose to interstitial glucose kinetics. The latter was considered by Knobbe *et al*. [77] who developed a five-state extended Kalman filter for the estimation of subcutaneous glucose levels, blood glucose levels, time lag between the sensor measured subcutaneous glucose and the blood glucose, time-rate-of-change of blood glucose level, and subcutaneous glucose sensor scale factor. Its performance was tested with data from four patients with diabetes, demonstrating the potential of this methodology to improve CGM accuracy. Facchinetti *et al*. further developed the strategy proposed by Knobbe *et al.* and proposed an ‘enhanced Bayesian calibration method (BCM)’ [78] based on an extended Kalman filter estimating interstitial glucose, plasma glucose and sensor sensitivity along time. A second order random walk model was proposed for describing plasma glucose; plasma-to-interstitial glucose relationship was described by a first order linear differential equation; and sensor sensitivity function was considered to be a triple integration of a zero-mean white noise. The method is intended to be used in cascade to any calibration algorithm built in commercial CGM, enhancing the monitor output for accuracy improvement. The method was validated on simulated data representative of diabetic subjects, and showed improved CGM accuracy as compared to the method of Knobbe *et al*. [77]. However, a drawback of this validation is the use of the same model of interstitial glucose and sensor sensitivity for data generation and state estimation, although in the first case a robustness analysis considering discrepancies in lag time estimation is conducted. Furthermore, as the authors acknowledge, application of the BCM to real data has two main limitations: first, it requires the knowledge of the variances of both state and measurement processes, which in real-life conditions are unknown; second, the existence of a *burn-in* period, considered as one day by the authors.

In summary, results from studies exploring new calibration strategies, suggest that the main limitation in current calibration algorithms may be linear regression. Unfortunately, complexity of the above mentioned methodologies [76–78] and the lack of its validation in a clinical context with data from prospective, controlled, randomized studies in diabetic subjects, do not still allow for their implementation in existing CGM devices. However, whatever the method used, it seems that consideration of dynamics of the physiological processes involved in glucose metabolism/kinetics may lead in the future to more accurate monitors.

## 5. Conclusions

Despite the huge amount of research in the field of CGM in the last 30 years, accuracy of devices currently available on the market is still suboptimal. Indeed, the final objective of technological research in diabetes is closed loop glycemic control. The complexity of building up an artificial pancreas system requires great quality of the input signal provided to the controller, *i.e.*, the continuous glucose registry. Improper calibration technique by the patient has been regarded as a major factor contributing to CGM inaccuracy. However, it is our opinion that refining CGM calibration algorithms is a priority of any artificial pancreas project. Indeed, linear regression methods do not permit full compensation of plasma-to-interstitium discrepancies during rapid changes in plasma glucose concentration and often result into erroneous predictions, especially in the hypoglycemic range. Better definition of the plasma to interstitial glucose dynamics is needed, under different metabolic conditions representative of the daily life of diabetic subjects. Inclusion of this information into new calibration algorithms has the potential for substantial improvement of CGM accuracy.

## Figures and Tables

**Table 1.** Devices currently available for home continuous glucose monitoring.

**Device**	**Company**	**Technique**	**Real-time**	**Associated with insulin pump**
CGMS iPro	Medtronic Minimed	Subcutaneous sensor	No	No
Guardian REAL-Time	Medtronic Minimed	Subcutaneous sensor	Yes	No
Paradigm REAL-Time	Medtronic Minimed	Subcutaneous sensor	Yes	Yes
Paradigm Veo	Medtronic Minimed	Subcutaneous sensor	Yes	Yes
SEVEN	Dexcom Inc.	Subcutaneous sensor	Yes	No
SEVEN Plus	Dexcom Inc.	Subcutaneous sensor	Yes	No
Freestyle Navigator	Abbott Inc.	Subcutaneous sensor	Yes	No

**Table 2.** Main characteristics of real-time calibration algorithms.

	**Medtronic**	**DexCom**	**Abbott**
Principle	Linear regression	Linear regression	Linear regression
Sensitivity change compensation	Recalibration when new blood glucose is obtained so as to minimization of MAD	Measurement of oxygen with auxiliary electrode and recalibration Substraction of sensor baseline signal	Sensitivity estimation through application of perturbation signals (response to different potentials)
Lag compensation	Weiner filter (no details provided by the manufacturer)	Linear/nonlinear regression used to predict values 15 min ahead	Estimation of glucose rate of change through filtering

**Table 3.** Accuracy of latest real-time continuous glucose monitors.

**Continuous glucose monitor**	**RAD (mean/median)**	**Gold standard**	**Source**
Abbott FreeStyle Navigator	12.8%/9.3%	Venous blood (YSI)	[72]
Medtronic Guardian REAL-Time	19.9%/16.7 %	Venous blood (YSI)	[73]
Medtronic Paradigm VEO	15.89%/11.56%	Capillary blood (glucometer)	[74]
Dexcom SEVEN Plus	15.9%/13%	Venous blood (YSI)	[40]
